# Professional-Facing Digital Health Solutions for the Care of Patients With Chronic Pain: Protocol for a Systematic Scoping Review

**DOI:** 10.2196/51311

**Published:** 2024-03-05

**Authors:** Haruno McCartney, Ashleigh Main, Maryam Ibrar, Harleen Kaur Rai, Natalie McFayden Weir, Roma Maguire

**Affiliations:** 1 Department of Computer and Information Sciences University of Strathclyde Glasgow United Kingdom; 2 Strathclyde Institute of Pharmacy and Biomedical Sciences University of Strathclyde Glasgow United Kingdom

**Keywords:** burden, chronic pain, clinician, digital health solution, digital health, digital solutions, eHealth, healthcare professional, mHealth, pain management, patient-facing, risk factor

## Abstract

**Background:**

Chronic pain is a highly prevalent condition and one of the most common reasons why people seek health care. As a result, chronic pain has a significant personal and economic burden. The COVID-19 pandemic has aggravated the situation for patients with chronic pain through increased risk factors (eg, anxiety or depression) as well as decreased access to health care. Digital health solutions to support people with chronic pain are becoming increasingly popular. Most of the research has focused on patient-facing digital health solutions, although it is clear that the involvement of health and care professionals is crucial in chronic pain care. Certainly, digital health solutions intended for the use of health and care professionals in the care of patients with chronic pain (ie, professional facing) exist, for example, for clinical decision support; however, no review has investigated the studies reporting these interventions.

**Objective:**

The overall aim of this scoping review is to identify the available professional-facing digital health solutions for the purpose of chronic pain management. The objectives of this review are to investigate the components, target populations, and user settings of the available professional-facing digital solutions; health and care professionals’ perspectives on using digital health solutions (if reported); the methods in which the digital health solutions are developed; and the outcomes of using professional-facing digital health solutions.

**Methods:**

Databases including MEDLINE, Embase, CINAHL, PsycInfo, and Inspec will be searched for studies reporting professional-facing digital health solutions for chronic pain care, using a comprehensive search strategy developed for each of the specific databases. A total of 2 independent reviewers will screen the titles and abstracts for review inclusion and then conduct full-text screening. Any conflicts in study inclusion will be resolved by a third reviewer at each stage of the screening process. Following data extraction and quality assessment, a qualitative content analysis of the results will be conducted. This review will identify the available professional-facing digital health solutions for chronic pain management. The results of this review are likely to be heterogeneous in terms of content (ie, the digital solutions will serve a variety of purposes, settings, target populations, etc) and methods (ie, experimental and nonexperimental designs).

**Results:**

The review is expected to finish in March 2024 and published in the summer of 2024.

**Conclusions:**

This protocol outlines the need for a scoping review to identify professional-facing digital health solutions for the management of chronic pain. Results from this review will contribute to the growing field of research into the utility of digital health for chronic pain management.

**International Registered Report Identifier (IRRID):**

DERR1-10.2196/51311

## Introduction

### Overview

Chronic pain is a highly prevalent condition, estimated to be affecting 20% to 30% of people worldwide [1,2]. Pain is defined by the International Association for the Study of Pain as an “unpleasant sensory and emotional experience associated with actual or potential tissue damage” [2]. Although there is some debate on the length of time an individual must experience pain for it to transition from “acute” to “chronic,” the *International Classification of Diseases, 11th Revision* defines chronic pain as pain lasting ≥3 months [3]. Chronic pain can also be further categorized into primary (no evident cause); secondary (as a result of an underlying condition); or in some cases, both [4,5].

Various approaches to chronic pain treatment exist, including pharmacological interventions (ie, analgesic medication from paracetamol to opioids) and nonpharmacological interventions (eg, physical therapy and psychological therapy such as cognitive behavioral therapy) [6]. The treatment of chronic pain can be extremely complex; mismanagement can lead to significant issues, for example, opioid dependency [7]. Working toward providing appropriate and effective treatment for patients with chronic pain is crucial to reducing the issues surrounding the mismanagement of chronic pain.

Pain is one of the most common reasons why people seek health care [1]. Due to the prevalence of the condition and its consequent impact on health care, chronic pain has a significant economic burden [1,8] with the estimated costs to the economy reaching billions in the United Kingdom [9,10] and other countries within Europe [11]. Furthermore, during the COVID-19 pandemic, health care resources were diverted from chronic pain management toward more emergency, COVID-19–related conditions [12]. The pandemic has exacerbated the challenges that patients with chronic pain may face; for example, social isolation and lockdown have led to reduced access to health care as well as increased anxiety or depression and reduced mobility, all of which are factors that may aggravate chronic pain symptoms [13-15]. Certainly, the pandemic has led to a reconsideration of traditional health care methods and has highlighted the importance of being flexible with novel methods of health care delivery [13,16]. There is a clear need for new innovations in chronic pain management [17] as a result of its economic burden, which has been further aggravated by the issues presented by the pandemic.

Digital health solutions may provide a unique opportunity to mitigate these challenges to chronic pain management cost-effectively [8,12]. There has been a recent rise in the number of digital solutions available for chronic pain management [14], such as applications for patient self-management [18] and digitally delivered physical therapy [19]. There is variation in the nomenclature of “digital health,” with definitions continuing to evolve [20]. Although there is no universally accepted definition for “digital health,” digital health refers to all digital, electronic, and computer technologies to improve health [21]; it is used synonymously with “eHealth” in much of the literature [21]. Indeed, terms such as “digital health,” “eHealth,” “mobile health (mHealth),” and “telemedicine or telehealth” have been used interchangeably [21]. The components of digital health are broad and may include mobile apps (mHealth), web applications, wearable technology, artificial intelligence, analytics, and telemedicine [20]. Digital solutions within health care provide many benefits, specifically to chronic pain management; they offer access to remote care and reduce the impact in some areas of health care service provision, for example, staff shortages and lack of resources [22]. It may also help in reducing waiting lists for care [23]. Now more than ever, this is important due to the challenges that health care systems face.

Previous reviews have focused on digital solutions for chronic pain [24-26] and for specific chronic pain conditions such as osteoarthritis and lower back pain [22,27]. Such reviews have focused particularly on the effect of digital solutions on patient outcomes, with many of the included interventions being patient-facing for the purpose of self-management. The results of these reviews show that digital solutions have a positive impact on patients with chronic pain, on outcomes such as pain intensity, quality of life, coping skills, and adherence to exercise [22,24-27].

There has been an emphasis on health and care professional involvement and contact as an important facilitator in the adoption of digital health solutions, as collaboration with key stakeholders and end-user groups is essential for the development of sustainable and usable systems [28]. Despite this, many digital solutions are developed and implemented without the involvement of health and care professionals [27]. Much of the literature also highlights the importance of multidisciplinary professional involvement in general chronic pain care [2,29,30]. Primary care clinicians are particularly essential in the process of chronic pain management [5,30]. Chronic pain management must go beyond self-management alone (through purely patient-facing solutions) and also involve multidisciplinary health and care professionals [2,5,29,30].

Previous studies on the perspectives of health and care professionals have also highlighted the potential of digital solutions as a useful tool for health and care professionals, that is, for education, patient-follow ups, etc [23]. Digital solutions targeting education for professionals may be particularly useful for chronic pain management, as negative attitudes and a lack of clinician knowledge are significant barriers to chronic pain care [31]. National guidelines state that it is essential for health and care professionals to have the best possible resources and support to manage patients with chronic pain [5], which could be facilitated by digital health solutions. Indeed, there are existing professional-facing digital solutions for the management of chronic pain, such as clinical decision support tools [32]. It is clear that there is a utility to digital health solutions for health and care professionals (ie, to provide education and other resources that adhere to guidelines for care) and that such applications exist; however, to our knowledge, no previous reviews have investigated the available professional-facing digital solutions for chronic pain.

Thus, the objective of this scoping review is to investigate the professional-facing digital solutions available for the management of chronic pain conditions.

### Review Question

This scoping review aims to answer the question: What research-based digital health solutions, specifically designed for the use of health and care professionals, are available for the management of chronic pain?

Therefore, the objectives of this scoping review are to investigate the following:

1. The components of existing professional-facing digital solutions for the management of chronic pain: (1) what are the user features, (2) what data do they collect, (3) are they integrated into larger systems or stand-alone (eg, within a system involving a patient-facing application), (4) security or privacy considerations, (5) target populations, and (6) settings (eg, pharmacy, hospital, or multisite adoption)
2. The frameworks and methods with which the digital solutions were designed and developed.
3. The outcomes measured and the effectiveness of the digital solutions, that is, implementation success and clinical effectiveness
4. Health and care professionals’ perceptions of the usability and usefulness of digital solutions in the management of chronic pain (if reported)

## Methods

The proposed scoping review will be conducted in accordance with the Joanna Briggs Institute methodology for scoping reviews [33] and will be reported according to the PRISMA-ScR (Preferred Reporting Items for Systematic Reviews and Meta-Analyses extension for Scoping Reviews) [34].

### Eligibility Criteria

The population, concept, and context (PCC) will be used to guide the assessment of studies for review inclusion.

#### Inclusion Criteria

##### Population

Studies will be included if the reported digital health solutions’ intended user demographic is health and care professionals directly involved in the care of patients (aged 18 years or older) with chronic pain. “Health and care professional” will refer to any professional who provides a health and care service, including, but not limited to, nurses, pharmacists, general practitioners, physiotherapists, other therapists, psychologists, and social care professionals. There will be no restrictions on the type of health and care profession of the participants.

##### Concept

The concept of interest for this review is digital health solutions (including, but not limited to, eHealth, mHealth, telehealth, web-based interventions, etc) intended to assist health and care professionals in the diagnosis and management of chronic pain. Any chronic pain-specific digital health solutions that are specifically designed for the end user to be health and care professionals will be considered in this review; therefore, the included solutions are likely to be heterogeneous in aim, user features, and functionality. This may include those intended for clinical decision support, education, remote patient monitoring, etc. Solutions may include mobile apps, web-based applications, or any other tools provided digitally.

Digital health solutions for the management of all types of chronic pain conditions will be considered, including chronic primary pain (eg, fibromyalgia, complex regional pain syndrome, and chronic primary headache), chronic secondary pain (eg, osteoarthritis and chronic pain secondary to cancer), or both [4,5]. Digital health solutions must be chronic pain specific, that is, for the management of a diagnosed chronic pain condition, with pain defined as “chronic” and lasting 3 or more months.

##### Context

There will be no restrictions on the context of this review. Contexts may include research and clinical settings, that is, any environment in which health and care professionals may be involved in the management of patients with chronic pain. Settings will likely include primary, secondary, and tertiary care settings (eg, pharmacies, general practitioners, and hospitals) and community settings (eg, residential facilities and the patient’s home).

#### Exclusion Criteria

Studies reporting digital health solutions intended for use by patients only (eg, for self-management purposes) without a professional-facing component will be excluded. Studies will also be excluded if the digital health solution is not specifically intended for the management of a chronic pain condition (eg, studies reporting communication between health care professionals and patients through telephone or videoconferencing [Zoom, Zoom Video Communications; Microsoft Teams, Microsoft Corporation; etc] only, without specific digital chronic pain support, or studies reporting management of chronic pain using a digital health solution designed for general health care, that is, electronic health records). Digital health solutions will not be considered if they support the care of acute or nonchronic pain, for example, nonspecific low back pain. Studies reporting digital health solutions for nonadult patients (<18 years old) with chronic pain will also be excluded. Other exclusion criteria include gray literature and studies not written in the English language.

#### Types of Sources

This scoping review will consider all types of study designs. This may include experimental studies on the effectiveness of professional-facing digital solutions for the management of chronic pain (eg, randomized controlled trials, before and after sequential design, etc) or nonexperimental studies on the development of digital solutions (eg, qualitative studies involving interviews). If only the abstract is available for a relevant study, the author will be contacted for further study information.

### Search Strategy

A comprehensive search strategy will be developed using subject headings specific to databases MEDLINE, Embase, CINAHL, PsycInfo, and Inspec (Multimedia Appendix 1). The search strategy will comprise three categories relevant to the PCC inclusion criteria: terms relating to (1) chronic pain (concept of interest), (2) digital health (concept of interest), and (3) health and care professionals (population). There will be no terms in the search strategy limiting the context, as there will be no restrictions on setting. Search terms used for chronic pain will be developed using terms used in the National Institute for Health and Care Excellence guidelines for the management of chronic pain [4]. Keywords and index terms from previous relevant literature will be used to form search terms for all categories within the search strategy. The search strategies will be reviewed by an academic librarian to gain assistance on database-specific guidelines and to ensure the searches capture as many papers fitting the eligibility criteria as possible. Backward-citation searches will also be conducted by reviewing the references of the included studies for relevant articles. Researchers and experts in the field will be contacted to inquire about studies that fit the inclusion criteria.

### Study or Source of Evidence Selection

Following the search and after removing duplicates, titles and abstracts will be screened by 2 independent reviewers against the inclusion criteria, with any disagreements resolved through discussion with a third independent reviewer. Full texts of potential studies fitting the inclusion criteria will then be reviewed again, in detail, by 2 independent reviewers. Reasons for exclusion will be recorded at the full-text reviewing stage and reported in the scoping review. Any disagreements that may arise at this stage will be resolved through discussion with a third independent reviewer. Screening and full-text review will be conducted on Covidence (Veritas Health Innovation Ltd).

### Data Extraction

Data will be extracted from the studies fitting the inclusion criteria by 2 independent reviewers using a data extraction tool developed for the purpose of this review (Multimedia Appendix 2). The data extraction tool will be developed from the Template for Intervention Description and Replication checklist [35], which focuses on the digital solution components, that is, the rationale of the intervention, user features, procedures, etc. Additional information specific to the review aims will also be collected, for example, participant demographic information and perspectives of health and care professional users (if reported). Data extraction will first be piloted by the 2 reviewers by extracting the data from 10% of the studies fitting the inclusion criteria together through discussion.

As the included studies are likely to be diverse in design (due to no restrictions being imposed on the type of source), quality assessment will be conducted using Quality Assessment for Diverse Studies [36]. Although it is not essential to conduct a quality assessment in a scoping review [33], ascertaining the overall quality of the studies included in this review will be beneficial, as the literature suggests research in digital health for chronic pain is of low quality [37].

### Data Analysis and Presentation

This scoping review will aim to conduct a qualitative content analysis of the results, that is, code the features of the digital health solutions used by the included studies into overall categories using NVivo (Lumivero, QSR International). If the results of the review are too heterogeneous for this, a narrative synthesis of the features of the digital health tools will be conducted. No analyses will be conducted to ascertain the overall effectiveness of professional-facing digital health solutions (as this is out of the scope of a scoping review). However, we will report the effectiveness of the individual digital health solutions as reported by each of the included studies in a narrative summary.

### Expected Results

This scoping review will identify the available professional-facing digital health solutions for the management of chronic pain, their components, user settings, and target populations; professionals’ perspectives on the use of digital health solutions; the methods in which the professional-facing digital health solutions are developed (ie, co-design framework used); and the outcomes of professional-facing digital health solutions. It is expected that the included studies will report on a variety of professional-facing digital health solutions. This may include systems for clinical decision support, symptom management, patient follow up, and educational tools. The outcomes of individual professional-facing digital solutions are also expected to be varied; they will likely have differing measures of effectiveness.

The types of studies fitting the inclusion criteria will most likely be heterogeneous in their methodology, as no restrictions have been imposed on design. Some included studies may report the early stages of developing the professional-facing digital solution (eg, nonexperimental studies such as those using interviews, surveys, and focus groups), while others may report on the effectiveness of the professional-facing digital solution (eg, experimental studies like randomized controlled trials). It is difficult to predict the quality assessment of these studies, at this stage.

The qualitative content analysis of the results will help to answer the review questions, that is, what are the user features, what data do they collect, are they integrated into larger systems or stand-alone, and what are their target population and user settings? If the included studies report on health and care professionals’ perspectives, for example, through interviews or surveys, qualitative analysis will also provide information on the health and care professionals’ perspectives on using professional-facing digital solutions.

## Results

The scoping review is expected to be finished by March 2024 and the first search will be implemented in July 2023. The results of the scoping review are expected to be published by the summer of 2024.

## Discussion

### Overview

To the best of our knowledge, this will be the first scoping review of professional-facing digital health solutions for the management of chronic pain. As we move further into a more digital age, there is a growing need for research into digital health and chronic pain care. This has been further emphasized by the challenges to care presented by the pandemic.

This scoping review will provide a foundation for further research into digital health solutions to make chronic pain care more efficient and innovative, from a health and care professional perspective. Results from this review will contribute to knowledge on the available professional-facing digital health solutions (including their components, user settings, target populations, development, and the perspectives of professionals on the use of such systems), as well as the gaps in the research on digital health solutions targeted toward health and care professionals (for example, areas of care that are not covered). As this is a scoping review, no conclusions can be drawn on the overall effectiveness of the professional-facing digital solutions, only the outcomes of the individual digital solutions as reported by the included studies.

We intend to conduct a qualitative content analysis to synthesize the results of this review. However, it is difficult to ascertain the method by which the included studies will report their findings (most likely extremely heterogeneous, given the broad inclusion criteria). Therefore, it may only be possible to conduct a narrative synthesis of the features of the professional-facing digital health solutions for chronic pain management. Either method of synthesizing the results of this review will produce valuable information on practices in chronic pain care and contribute knowledge to the field of digital health research.

### Patient-Facing Versus Professional-Facing Digital Health Solutions for Chronic Pain Management

Although previous reviews have not focused on digital health solutions intended for use by health and care professionals, the literature supports the effectiveness of digital health solutions for the management of chronic pain. Recent systematic reviews suggest that mHealth interventions for chronic pain have a positive effect on patient outcomes (eg, pain intensity, quality of life, and functional ability) [25,26]. However, the literature also suggests that the quality of studies on digital health solutions for chronic pain is subpar, with Moreno-Ligero and colleagues [25] reporting “medium” quality for the majority of their included studies. This further highlights the need for a quality assessment to be conducted in this scoping review, to ascertain the quality of the studies on professional-facing digital solutions for chronic pain management.

Similarly, previous studies reporting on health and care professionals’ perspectives have focused on patient-facing digital health solutions for chronic pain management, that is, self-management interventions. Varsi and colleagues [23] emphasized the need for a comprehensive treatment approach to chronic pain care of which digital health solutions could provide if factors such as health care provider involvement, timely support, and follow up are also considered. Professional-facing digital health solutions may be one of the ways in which the latter factors could be incorporated into a more comprehensive system for managing chronic pain. Additionally, health and care professional involvement is required, not only in the use of digital health solutions but also in their development; many studies reporting digital health solutions for chronic pain management were designed without stakeholder involvement [23] and developed without using co-design methods or any form of user evaluation. This is also particularly essential for professional-facing digital health solutions and will be addressed in this review.

### Strengths and Limitations

A significant strength of this scoping review is the systematic method of conducting the searches. A specialized academic librarian will aid in the development of comprehensive and individualized search strategies (Multimedia Appendix 1). Search terms will be formulated using specific subject headings for each database (eg, Medical Subject Headings for MEDLINE) as well as keywords taken from previous similar reviews. Search terms to describe chronic pain will be developed from terms used in the National Institute for Health and Care Excellence guidelines for the management of chronic pain [4]. A total of 2 blinded reviewers will independently complete all steps of the reviewing process, and a third reviewer will resolve any disagreements. Due to this systematic process, this review is likely to include most of the relevant studies that fit the inclusion criteria. Furthermore, this scoping review will follow the Joanna Briggs Institute scoping review manual as well as the PRISMA-ScR checklist.

The findings of this review will be limited to those published in the English language; therefore, there is a possibility that some professional-facing digital health solutions developed and reported in non-English–speaking countries will be missed by this review. It is important to consider that, as guidelines for chronic pain care and digital health differ between countries, jurisdiction of development and implementation may be a significant factor in the type of digital health solutions reported in this review. Furthermore, as some digital health solutions are developed commercially and do not have an academic foundation, the search of academic databases (without a market review, eg, app store search) may not have captured all professional-facing digital health solutions for chronic pain management. However, the quality of digital health solutions is largely dependent on their scientific foundation, which is facilitated by academic development, and nonevidence-based professional digital health solutions are not likely to be useful or realistically implemented in clinical chronic pain care settings. In order to find all available digital health solutions, it would be useful to communicate with health and care professionals currently involved in chronic pain care. Future studies may benefit from conducting interviews with health and care professionals to ascertain what digital health solutions are currently implemented in clinical settings and the benefits or drawbacks of such systems in the care of patients with chronic pain.

### Conclusion

This protocol outlines the need for a scoping review to identify professional-facing digital health solutions for the management of chronic pain. The results of this review will highlight the available digital health solutions available for health and care professionals to use specifically for chronic pain care and provide information regarding the system’s components, user settings, specific target populations, and development. This scoping review will be systematically conducted to ensure most of the relevant literature is included; however, some professional-facing digital health solutions may be missed if they are developed commercially (without academic publication) or not reported in the English language. Findings from this review will contribute to the ever-growing field of digital health research and provide further information on how digital health can improve chronic pain care.

## Appendix

Multimedia Appendix 1 Search strategies.

Multimedia Appendix 2 Data extraction template.

## Abbreviations

mHealth: mobile health
PCC: population, concept, and context
PRISMA-ScR: Preferred Reporting Items for Systematic reviews and Meta-Analyses extension for Scoping Reviews

## Data Availability

Data sharing is not applicable to this article as it is a protocol and no data sets have been generated or analyzed yet.
