# Supplementary material for: Professional-Facing Digital Health Solutions for the Care of Patients With Chronic Pain: Protocol for a Systematic Scoping Review
Source: JMIR Res Protoc. 2024 Mar 5;13:e51311. doi: 10.2196/51311 (PMC10951835; doi:10.2196/51311)
Supplement: Multimedia Appendix 1 [file resprot_v13i1e51311_app1.docx]

# Search strategies.

## MEDLINE search strategy (OVID)

1. Chronic pain/
2. Cancer pain/
3. (chronic adj2 (pain OR primary pain OR secondary pain OR migraine OR headache* OR ache)).mp
4. (Persistent pain* OR cancer pain OR fibromyalgia OR back pain* OR sciatica OR arthritis OR rheumatoid arthritis OR osteoarthritis OR spondylarthritis OR endometriosis OR neuropathic pain* OR irritable bowel syndrome OR complex regional pain syndrome OR orofacial pain OR chronic primary visceral pain OR joint pain* OR musculoskeletal pain*).mp.
5. OR/1-4
6. Telemedicine/
7. (eHealth OR mHealth).mp.
8. (electronic OR web* OR digital OR mobile OR tele OR app OR smartphone OR internet adj3 (medicine OR health OR clinic* OR decision support)).mp.
9. OR/6-8
10. Health personnel/
11. Primary healthcare/
12. Health services/
13. Social support/
14. (healthcare profession* OR clinician-facing OR professional-facing OR professional portal OR healthcare provider* OR health care* OR social care* OR multidisciplinary clinic* OR pharmac* OR clinic*).mp.
15. OR/10-14
16. AND/5, 9, 15
17. Limit 16 to English Language

## EMBASE (OVID)

1. Chronic pain/
2. Cancer pain/
3. (Chronic pain OR chronic primary pain OR chronic secondary pain OR chronic migraine OR cancer pain OR persistent pain* or fibromyalgia or back pain* or sciatica or arthritis or rheumatoid arthritis or osteoarthritis or spondylarthritis or endometriosis or neuropathic pain* or irritable bowel syndrome or complex regional pain syndrome or orofacial pain or chronic primary visceral pain or joint pain* or musculoskeletal pain*).mp.
4. OR/1-3
5. Telehealth/
6. (eHealth OR mHealth).mp.
7. (electronic OR web* OR digital OR mobile OR tele OR app OR smartphone OR internet adj3 (medicine OR health OR clinic* OR decision support)).mp.
8. OR/5-7
9. Health personnel/
10. Health care delivery/
11. Social care/
12. (healthcare profession* OR clinician-facing OR professional-facing OR professional portal OR healthcare provider* OR health care* OR social care* OR multidisciplinary clinic* OR pharmac* OR clinic*).mp.
13. OR/9-12
14. AND/4, 8, 13
15. Limit 14 to English Language

## CINAHL (EBSCOhost)

(MH “Chronic pain+”) OR (TI “chronic pain” OR TI “chronic primary pain” OR TI “chronic secondary pain” OR TI “chronic migraine” OR TI “cancer pain” OR TI “persistent pain” or TI “fibromyalgia” OR TI “back pain” OR TI “sciatica” OR TI “arthritis” OR TI “rheumatoid arthritis” OR TI “osteoarthritis” OR TI “spondylarthritis” OR TI “endometriosis” OR TI “neuropathic pain” OR TI “irritable bowel syndrome” OR TI “complex regional pain syndrome” OR TI “orofacial pain” OR TI “chronic primary visceral pain” OR TI “joint pain” OR TI “musculoskeletal pain”) OR (AB “chronic pain” OR AB “chronic primary pain” OR AB “chronic secondary pain” OR AB “chronic migraine” OR AB “cancer pain” OR AB “persistent pain” or AB “fibromyalgia” OR AB “back pain” OR AB “sciatica” OR AB “arthritis” OR AB “rheumatoid arthritis” OR AB “osteoarthritis” OR AB “spondylarthritis” OR AB “endometriosis” OR AB “neuropathic pain” OR AB “irritable bowel syndrome” OR AB “complex regional pain syndrome” OR AB “orofacial pain” OR AB “chronic primary visceral pain” OR AB “joint pain” OR AB “musculoskeletal pain”) OR (SU “chronic pain” OR SU “chronic primary pain” OR SU “chronic secondary pain” OR SU “chronic migraine” OR SU “cancer pain” OR SU “persistent pain” or SU “fibromyalgia” OR SU “back pain” OR SU “sciatica” OR SU “arthritis” OR SU “rheumatoid arthritis” OR SU “osteoarthritis” OR SU “spondylarthritis” OR SU “endometriosis” OR SU “neuropathic pain” OR SU “irritable bowel syndrome” OR SU “complex regional pain syndrome” OR SU “orofacial pain” OR SU “chronic primary visceral pain” OR SU “joint pain” OR SU “musculoskeletal pain”)

AND

(MH “Digital health+”) OR (MH “Decision support systems, clinical”) OR (TI “digital health” OR TI “mHealth” OR TI “eHealth” OR TI “electronic health” OR TI “mobile health” OR TI “telehealth” OR TI “mobile application” OR TI “app”) OR (AB “digital health” OR AB “mHealth” OR AB “eHealth” OR AB “electronic health” OR AB “mobile health” OR AB “telehealth” OR AB “mobile application” OR AB “app”) OR (SU “digital health” OR SU “mHealth” OR SU “eHealth” OR SU “electronic health” OR SU “mobile health” OR SU “telehealth” OR SU “mobile application” OR SU “app”)

AND

(MH “Health personnel+”) OR (TI “clinician-facing” OR TI “professional-facing” OR TI “professional portal” OR TI “clinician” OR TI “healthcare professional” OR TI “healthcare provider” OR TI “primary healthcare” OR TI “multidisciplinary clinic*” OR TI “pharmac*” OR TI “nurs*” OR TI “social care” OR TI “health care”) OR (AB “clinician-facing” OR AB “professional-facing” OR AB “professional portal” OR AB “clinician” OR AB “healthcare professional” OR AB “healthcare provider” OR AB “primary healthcare” OR AB “multidisciplinary clinic*” OR AB “pharmac*” OR AB “nurs*” OR AB “social care” OR AB “health care”) OR (SU “clinician-facing” OR SU “professional-facing” OR SU “professional portal” OR SU “clinician” OR SU “healthcare professional” OR SU “healthcare provider” OR SU “primary healthcare” OR SU “multidisciplinary clinic*” OR SU “pharmac*” OR SU “nurs*” OR SU “social care” OR SU “health care”)

Limiters = English Language

## PsycInfo (OVID)

1. Chronic pain/
2. (chronic adj2 (pain OR primary pain OR secondary pain OR migraine OR headache* OR ache)).mp.
3. (Persistent pain* OR cancer pain OR fibromyalgia OR back pain* OR sciatica OR arthritis OR rheumatoid arthritis OR osteoarthritis OR spondylarthritis OR endometriosis OR neuropathic pain* OR irritable bowel syndrome OR complex regional pain syndrome OR orofacial pain OR chronic primary visceral pain OR joint pain* OR musculoskeletal pain*).mp.
4. OR/1-3
5. Telemedicine/
6. Digital health resources/
7. Electronic health services/
8. (eHealth OR mHealth).mp.
9. (electronic OR web* OR digital OR mobile OR tele OR app OR smartphone OR internet adj3 (medicine OR health OR clinic* OR decision support)).mp.
10. OR/5-9
11. Health personnel/
12. Clinicians/
13. (healthcare profession* OR clinician-facing OR professional-facing OR professional portal OR healthcare provider* OR health care* OR social care* OR multidisciplinary clinic* OR pharmac* OR clinic*).mp.
14. OR/11-13
15. AND/4, 10, 14
16. Limit 15 to English Language

## INSPEC (includes ACM)

SUBJECT/TITLE/ABSTRACT

Chronic pain OR chronic primary pain OR chronic secondary pain OR chronic migraine OR cancer pain OR persistent pain or fibromyalgia or back pain or sciatica or arthritis or rheumatoid arthritis or osteoarthritis or spondylarthritis or endometriosis or neuropathic pain or irritable bowel syndrome or complex regional pain syndrome or orofacial pain or chronic primary visceral pain or joint pain or musculoskeletal pain

AND

Digital health OR eHealth OR mHealth OR telehealth OR telemedicine OR electronic health OR mobile health OR clinical decision support OR mobile application OR app

AND

Clinician OR healthcare professional OR healthcare provider OR primary healthcare OR health care OR social care OR clinician-facing OR professional-facing OR professional portal OR health personnel OR multidisciplinary clinic* OR pharmac*

Limiters = English Language
